# Tracking family medicine graduates. Where do they go, what services do they provide and whom do they see?

**DOI:** 10.1186/1471-2296-13-26

**Published:** 2012-03-28

**Authors:** R Liisa Jaakkimainen, Susan E Schultz, Richard H Glazier, Caroline Abrahams, Sarita Verma

**Affiliations:** 1 Department of Family and Community Medicine, University of Toronto, Toronto, ON, Canada; 2 Institute for Clinical Evaluative Sciences, 2075 Bayview Ave, G Wing, Toronto, ON M4N 3M5, Canada; 3 Department of Family and Community Medicine, Sunnybrook Health Sciences Centre, Toronto, ON, Canada; 4 Department of Family and Community Medicine, St. Michael's Hospital, Toronto, ON, Canada; 5 Post Graduate Medical Education Office, University of Toronto, Toronto, ON, Canada

## Abstract

**Background:**

There are continued concerns over an adequate supply of family physicians (FPs) practicing in Canada. While most resource planning has focused on intake into postgraduate education, less information is available on what postgraduate medical training yields. We therefore undertook a study of Family Medicine (FM) graduates from the University of Toronto (U of T) to determine the type of information for physician resource planning that may come from tracking FM graduates using health administrative data. This study compared three cohorts of FM graduates over a 10 year period of time and it also compared FM graduates to all Ontario practicing FPs in 2005/06. The objectives for tracking the three cohorts of FM graduates were to: 1) describe where FM graduates practice in the province 2) examine the impact of a policy introduced to influence the distribution of new FM graduates in the province 3) describe the services provided by FM graduates and 4) compare workload measures. The objectives for the comparison of FM graduates to all practicing FPs in 2005/06 were to: 1) describe the patient population served by FM graduates, 2) compare workload of FM graduates to all practicing FPs.

**Methods:**

The study cohort consisted of all U of T FM postgraduate trainees who started and completed their training between 1993 and 2003. This study was a descriptive record linkage study whereby postgraduate information for FM graduates was linked to provincial health administrative data. Comprehensiveness of care indicators and workload measures based on administrative data where determined for the study cohort.

**Results:**

From 1993 to 2003 there were 857 University of Toronto FM graduates. While the majority of U of T FM graduates practice in Toronto or the surrounding Greater Toronto Area, there are FM graduates from U of T practicing in every region in Ontario, Canada. The proportion of FM graduates undertaking further emergency training had doubled from 3.6% to 7.8%. From 1993 to 2003, a higher proportion of the most recent FM graduates did hospital visits, emergency room care and a lower proportion undertook home visits. Male FM graduates appear to have had higher workloads compared with female FM graduates, though the difference between them was decreasing over time. A 1997 policy initiative to discount fees paid to new FPs practicing in areas deemed over supplied did result in a decrease in the proportion of FM graduates practicing in metropolitan areas.

**Conclusions:**

We were able to profile the practices of FM graduates using existing and routinely collected population-based health administrative data. Further work tracking FM graduates could be helpful for physician resource forecasting and in examining the impact of policies on family medicine practice.

## Background

Family physicians (FPs) are viewed as the gatekeepers to the Canadian healthcare system. They provide comprehensive health care, continuity of care and coordinate care between different health care providers and health care sectors. Thus the Canadian government has been seeking strategies to address the concern that many people do not have a regular family physician [1,2]. In the late 1990s, there was a decline in the number of medical graduates in Canada applying to Family Medicine (FM) residency positions [3]. A similar decrease in the popularity of FM post-graduate training was seen in the US, UK and Australia [4-6]. While this trend may be changing in Canada, a strong family medicine workforce is needed to ensure access to the healthcare system and to face challenges such as the expansion of newer chronic disease management initiatives [7,8].

Most physician resource planning in Canada has focused on intake into FM training. Recent information identifies specific demographic (for example older trainees and those in committed relationships) and attitudinal factors (for example a desire for varied scope of practice, societal orientation, lower preference for medical versus social problems) at entry into medical school which influence choosing FM as a career [9]. This is important as in Canada postgraduate training is the most important input to physician supply. Not only do Canadian medical graduates require postgraduate training in order to become licensed in Ontario, but the majority of International Medical Graduates (IMGs) require full or partial residency training in order to be eligible to practice. A review of the influence of education and training of FPs in Australia found little research on the influence of FM training programs on post-educational clinical practice [10]. There has been less information on what postgraduate medical training in Canada yields and its potential impact on physician supply.

The scope of practice provided by FM graduates and their overall workload after they have completed a residency program has been examined in the US and Australia [11-15]. Other studies have examined the outcomes of certain specialized training programs within the FM curriculum, such as emergency medicine or rural health care, on their FM graduates [16-18]. Most of the information for these tracking studies has come from surveys of the FM graduates. Less is known about the types of patients cared for by FM graduates in any jurisdiction.

The University of Toronto (U of T) is the largest medical school in Canada. Approximately 20% of new FP and specialist physicians in Canada are graduates of the U of T postgraduate residency program [19]. In 2006, 33% of new Ontario trained FPs exited from U of T [19]. We therefore undertook a record linkage study using Ontario health administrative data for FM graduates from U of T. The overall purpose of generating this information is to determine the type of information for physician resource planning that may come from tracking FM graduates using health administrative data.

This study compared three cohorts of FM graduates over a 10 year period of time and it also compared FM graduates to all Ontario practicing FPs in 2005/06. The aim of this study was to find out where U of T FM graduates practice in Ontario and what services they provide. The objectives for tracking the three cohorts of FM graduates were to: 1) describe where FM graduates practice in the province 2) examine the impact of a policy introduced to influence the distribution of new FM graduates in the province 3) describe the services provided by FM graduates and 4) compare workload measures. Another aim of this study was to compare U of T FM graduates to practicing FPs in Ontario. The objectives for the comparison of FM graduates to all practicing FPs in 2005/06 were to: 1) describe the patient population served by FM graduates, 2) compare workload of FM graduates to all practicing FPs.

## Methods

### Study design

This is a cross-sectional study of three cohorts of FM graduates and FM graduates in 2005/06 using health administrative data based measures of patient demographics, health care services provided, workload measures and location of practice.

### Data sources

Family Medicine (FM) graduates: U of T FM graduates were identified using the U of T POstgraduate Web Evaluation and Registration system (POWER). POWER is a fully web-based postgraduate trainee information system designed by Knowledge4You and used exclusively by the U of T postgraduate medical program. POWER was introduced at U of T in 2004, with historical individual level registration records imported into the database back to the early 1980's. Individual postgraduate trainees (residents and fellows) are tracked according to unique student information numbers and registration numbers with the College of Physicians and Surgeons of Ontario (CPSO). All postgraduate trainees are registered in POWER and an ongoing record is maintained that includes: training level, program, source of funding, legal status, clinical rotations and change in status due to leaves of absence, research fellowships and exit from training. Postgraduate trainees consent to storing and retrieval of their registration data for research purposes as part of their Letter of Appointment with the University of Toronto.

### Practicing physicians

The Institute for Clinical Evaluative Sciences (ICES) Physician Database (IPDB) is constructed using data from the Ontario Physician Human Resource Data Centre (OPHRDC) file of physicians in active practice in Ontario, the Ontario Health Insurance Plan (OHIP) Corporate Provider Database (CPDB) and the OHIP file of physician billings. It is completely anonymized, with all individual identifiers either removed or encrypted. It includes records for all physicians in practice in Ontario for the years 1992 to 2009.

### Health administrative data

OHIP billings were used to link patients with physicians. Information on patients' age, sex and geographic location was provided by the Registered Persons Database (RPDB). Physician group affiliations were identified in the Client Agency Program Enrolment (CAPE) database of patient enrollments with primary care groups and the OHIP CPDB. Primary care reform became active in Ontario, Canada starting in 2003. The CAPE database identifies patients belonging to the newer primary care models of Family Health Groups (FHGS), Family Health Networks (FHNs), Family Health Organizations (FHOs) and Family Health Teams (FHTs). Prior to 2003, primary care groups mostly consistent of Health Service Organizations (FHOs) and other salary or capitation-based practices. Patients attending Community Health Centres (CHCs) are not included in the CAPE database. Currently in Ontario Canada, there are 73 CHCs with less than 5% of Ontario FPs practicing in a CHC [20].

### Definitions

#### Study cohort

The study cohort was extracted from POWER and consisted of all U of T postgraduate FM trainees who started their training in first year (PGY1) and exited training between 1993 and 2003. Trainees on temporary work authorizations (visa trainees) were excluded. The data from POWER included a trainee's residency program, start and end dates and training level at the beginning of training and training level at the end.

To examine changes over time, we examined three cohorts of FM graduates. These cohorts were defined by FM graduates exit year of graduation (1993 to 1996, 1997 to 1999 and 2000 to 2003). We also examined FM graduates practicing in 2005/06 and compared them with all FPs practicing in Ontario in 2005/06.

#### Record linkage

The unique identifier used for each FM graduate was their CPSO registration number. The data were extracted from POWER and sent to ICES where the data were cleaned and anonymized. Anonymization involved stripping off the names and addresses and then encrypting their CPSO registration number using the ICES encryption algorithm. The data from POWER was then linked to the IPDB on the encrypted CPSO number. Any FM trainee whose number did not link was assumed not to be in active practice in Ontario.

#### Variable definitions

Because patients often see more than one FP in a year, a FP's patient population consisted of all the patients for whom they provided the majority of their care, based on their OHIP billings. The majority rule assignment has been used in several other studies examining primary health care in Ontario [21,22]. Each FP's main practice venue was defined using information from the CPDB that identifies the types of groups physicians are affiliated with. The main practice venue is the venue where the physician delivered most of his or her care. In the case of primary care groups, group information had to first be derived from the CAPE database and then added to the OHIP data.

Patient acuity reflects the severity of an individual's health conditions. For this study, the Johns Hopkins Adjusted Clinical Group (ACG) case-mix system, was used as a measure of patient acuity [23]. The Johns Hopkins ACG system is based on patients' diagnoses from physician visits and hospital admissions. All diagnoses are assigned to one of 32 diagnosis clusters known as Aggregated Diagnosis Groups (ADGs). In this classification, International Classification of Disease (ICD) codes within the same ADG are similar in terms of both clinical criteria and expected need for healthcare resources. Just as individuals may have multiple ICD diagnosis codes, they may have multiple ADGs (up to 32). The number of ADGs a person had was summed and then grouped into acuity levels. Those with the greatest number of ADGs (in this case 10 or more) are the sickest and require the most healthcare resources.

The Ontario Ministry of Health and Long Term Care (MOHLTC) divided the province into 14 regions or Local Health Integration Networks (LHINs). The LHINs were developed to locally organize, plan, fund and integrate health services such as hospitals and community health services. To describe where practices were located, physicians and patients were assigned to LHINs using the postal code conversion file (PCCF) developed by Statistics Canada [24].

A proxy measure for socioeconomic status was based on the ranking of each neighbourhood's average household income compared to all other neighbourhoods in a given municipality [25]. These neighbourhood income quintiles were developed by Statistics Canada and have been used in multiple health administrative studies in Canada.

Workload was measured by calculating a full-time equivalent (FTE). The FTE measure is based on the OHIP billings of physicians, both using fee-for-service (FFS) billings and shadow-billings, acting as a proxy measure of services rendered. FTE was based on the overall distribution of billings, whereby all physicians whose total adjusted billings fell between the 40th and 60th percentile are said to be working the equivalent of one FTE [1,26]. FPs in some primary care delivery models submit shadow billings. They are identical to FFS billing except there is no actual payment. However, there may be concern that shadow billings are lower that FFS physician billings leading to an underestimation of services provided by FPs who shadow bill. Therefore for the workload analysis, only physician who were mainly FFS were included. In 2005, this represented more than 90% of FPs in Ontario [27].

#### Analysis

Two comparisons were undertaken. We compared the three FM graduate cohorts by their exit year and we examined FM graduates who were practicing in 2005/06 and compared them to all FPs practicing in Ontario in 2005/06. Comparisons were tested for statistical significance using a two-sample *t*-test (with a *p *< 0.05) and by calculating 95% confidence intervals using SAS software [28].

This study was a collaborative effort between the University of Toronto Postgraduate Medical (PGME) Education Office and the Institute for Clinical Evaluative Sciences. It was approved by the Research Ethics Boards of both the University of Toronto and Sunnybrook Health Sciences Centre.

## Results

### FM graduates

The total number of FM graduates who exited training at U of T between 1993 and 2003 was 857 (Table 1). There were 768 (89.6% of the total) FM graduates in practice in Ontario 3 years after completing their training. There were 728 (84.9% of the total) FM graduates who billed the OHIP in fiscal 2005/06.

**Table 1:** Demographic characteristic and undergraduate medical training of University of Toronto Family Medicine Graduates

	Exit cohort						All Cohorts	
	1993-1996		1997-1999		2000-2003			
	N	%	N	%	N	%	N	%
**Total in cohort**	**303**	**100**	**248**	**100**	**306**	**100**	**857**	**100**
Age at entry into PG Training								
< 25 years	62	20.5	44	17.7	28	9.1	134	15.6
25-26 years	107	35.3	82	33.1	121	39.5	310	36.2
27-29 years	69	22.8	48	19.3	88	28.8	205	23.9
30-34 years	40	13.2	39	15.7	34	11.1	113	13.2
35-39 years	17	5.6	19	7.7	21	6.9	57	6.6
40 or older	8	2.6	16	6.4	14	4.6	38	4.4
Mean age (years)	27.6		28.8		28.3		28.2	
Sex								
Male	176	58.1	111	44.8	122	39.9	409	47.7
Female	127	41.9	137	55.2	184	60.1	448	52.3
Undergraduate Medical School								
Toronto	176	58.1	121	48.8	118	38.6	415	48.4
Other Ontario	69	22.8	48	19.3	98	32.0	215	25.1
Other Canada or U.S.	27	8.9	38	15.3	57	18.6	122	14.2
Outside Canada/U.S.	31	10.2	41	16.5	33	10.8	105	12.2

The overall proportion of FM graduates was 30.6% of all U of T medical graduates. This proportion declined from 33.3% in the exit years 1993 to 1996, to 28.9% in exit years 1997 to 1999 and 29.6% in the exit years 2000 to 2003. The proportion of FM graduates choosing the emergency medicine option doubled over the 10 year time frame from 3.6% to 7.8%. Overall, 12.2% of FM graduates received their undergraduate medical training outside of Canada or the United States.

### FM graduates exit year cohorts

**Figure 1:**
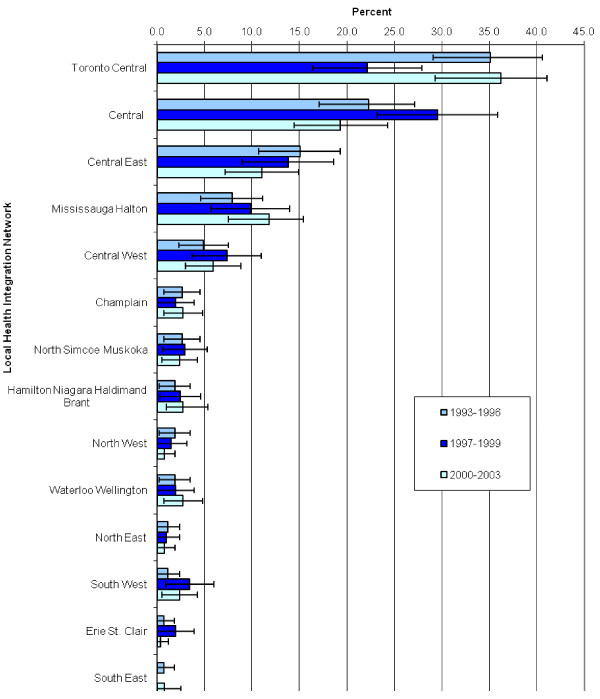
**Practice location of University of Toronto Family Medicine graduate exit cohorts, by Local Health Integration Network**.

Most U of T FM graduates practiced in Toronto or the surrounding Greater Toronto Area (Figure 1). However, there were FM graduates from U of T practicing in every region in Ontario, Canada. For FM graduates exiting their training between 1997 and 1999, there was a statistically significant drop in the proportion of FM graduates who practicing in the Toronto Central area. While there was an increased proportion in the adjacent Central LHIN, this was not statistically significant.

**Figure 2:**
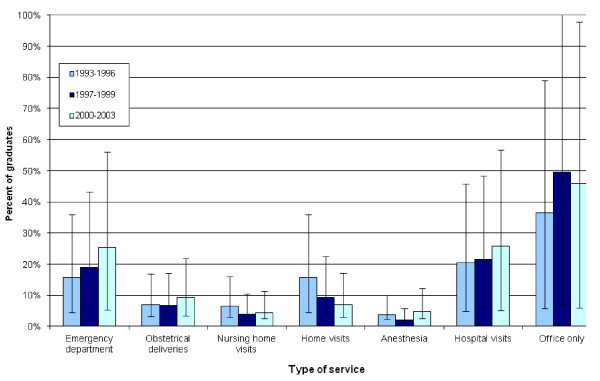
**Office versus out-of-office services provided by University of Toronto Family Medicine graduate exit cohorts**.

Figure 2 looks at the types of out-of-office services provided by FM graduates over time. A higher proportion of the most recent cohort of FM graduates worked in the Emergency Department (ED), did obstetrical deliveries and hospital visits. On the other hand, a higher proportion of the earliest cohort did home visits. The middle cohort had the highest proportion of members who had an office-based practice only. However, none of these trends were statistically significant.

Table 2 compares the head count, FTEs and ratio of FTE/head count for FM graduates who exited from residency training at U of T at all three different times. Female FM graduates had a statistically significant lower FTE/head count ratio than their male counterparts. With respect to male FPs, graduates from the earlier cohort seem to work slightly more. The FTE/head count ratio gap between male and female FM graduates narrowed over time.

**Table 2:** Number (head count), full-time equivalents (FTEs) and Ratio of University of Toronto Family Medicine Graduates, by sex, and exit cohort

Exit cohort	Measure	Female	Male
1993-1996	Head count	99	140
	FTE	84	167
	Ratio	0.85 (0.77, 0.92)	1.19 (1.13, 1.26)
1997-1999	Head count	106	85
	FTE	87	100
	Ratio	0.82 (0.74, 0.89)	1.18 (1.09, 1.25)
2000-2003	Head count	153	111
	FTE	119	116
	Ratio	0.78 (0.72, 0.84)	1.05 (0.97, 1.13)

### FM graduates in 2005/06

**Figure 3:**
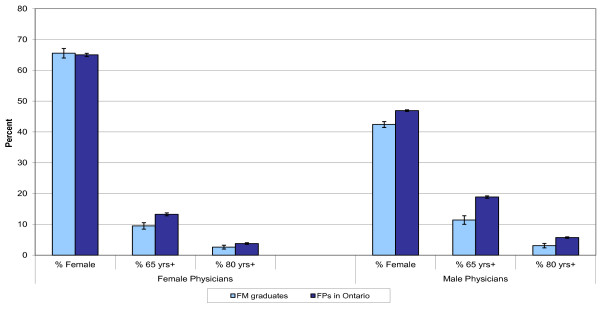
**Patient demographic characteristics for University of Toronto Family Medicine Graduates and all Family Physicians in Ontario, 2005/06**.

Figure 3 describes the demographic characteristics of patients served by FM graduates compared to all FPs practicing in Ontario in 2005/06. Female physicians had a much higher proportion of female patients than male physicians. In 2005/06, male FPs had a larger proportion of patients over 65 years of age compared to female FPs in Ontario, though this difference was not statistically significant amongst FM graduates. The U of T FM graduates had a lower proportion of older patients in their practice compared with all FPs practicing in Ontario.

In 2005/06, 40% of U of T FM graduates were in a solo practice as their main practice venue, 28% in a primary care group, 18% in a community based group, 5% in an academic health centre and 5% in an emergency department.

The number of ADGs per patient, per practice was used as a measure for patient acuity, with a higher number of ADGs representing higher patient comorbidity. In 2005/06, 8% of patients in the practices of U of T FM graduates had no ADGs (no comorbidity). However, 8% of patients of U of T FM graduates belonged to 1 ADG, 45% to 2-5 ADGs and 39% to over 6 ADGs (high comorbidity).

**Figure 4:**
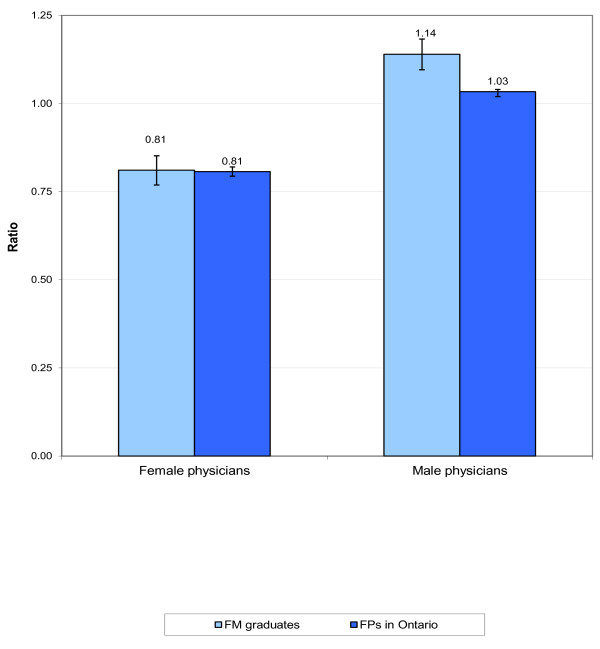
**Ratio of full-time equivalents to head count, University of Toronto Family Medicine graduates and all Family Physicians in Ontario in 2005/06**.

The proxy measure for SES was neighbourhood income quintiles. In 2005/06 17% of the patients of FM graduates were from the poorest neighbourhoods compared to 22% of patients who came from the wealthiest neighbourhoods. There was no statistical difference in comparison to postgraduates from other programs at U of T (data not shown). Figure 4 compares the FTE/head count ratio of FM U of T graduates to all other FM physicians practicing in Ontario in 2005/06. There was very little difference between the two groups for female family physicians. However, male FM U of T graduates appeared to have higher workloads than male FM physicians practicing in Ontario in 2005/06.

## Discussion

From 1993 to 2003 there was a slight decrease in the proportion of FM graduates from U of T. This decline in FM parallels the decline in the recruitment of medical students into family medicine programs in Ontario, Canada, as well in other countries such as the US, Australia and the UK [3,29-33]. However, the majority of FM physicians who completed postgraduate training continued to practice FM years after graduating. This is slightly better than the estimate that 1 in 9 Canadian physicians from each graduating class joined the US physician workforce between 2004 and 2006 [34]. It is similar to a 1991 UK study found that 13.0% of new entrant GPs left training after two years [35]. This is important information for physician resource planning, since there is little information about the retention of new FM graduates in the province. In Ontario, there are opportunities for practicing physicians to re-enter postgraduate training, usually into more specialized medical areas [36]. Our data may miss some of these trainees because re-entry opportunities were not made available until 1997 and they have not been in their new specialty for at least 3 years. However, it does appear that, at the most, only 1% of U of T FM graduates took advantage of these retraining programs.

Canada started to reforms its primary care delivery system after the release of the Romanow report in 2002 [37]. Each province in Canada approached primary care reform in different ways, with Ontario focusing on the implementation of newer primary care delivery models [38]. Currently, there has been a reversal in the decline of medical graduates choosing FM postgraduate positions in almost all areas of Canada. This has been seen amongst U of T FM programs and within most other FM programs across Canada [39,40]. The retention of U of T FM graduates continuing to practice FM within Canada is also similar to FM graduates from other medical schools in Canada [41,42]. While no formal evaluations exist to account for the increasing trend of medical graduates choosing FM across Canada, primary care reform is likely to have been a major influence.

While only 30% of FM graduates appeared to be practicing in a primary care group, this proportion corresponds to Ontario estimates for 2005 [43]. This analysis was done at the beginning of primary care reform in Ontario, Canada. It is likely to be higher now since well over half the family physicians in Ontario had joined one of the newer primary care groups by 2007 [44].

Looking at the out-of-office versus office services provided by FM graduates, the more recent graduates were more likely than their earlier graduating counterparts to be working in the Emergency Department in 2005/06. This has been seen in other programs in Ontario, Canada and also in the US [15,45]. However, it contrasts with a decline in emergency room care provided by FM graduates from the University of Missouri [13]. This may also reflect the development and expansion of the CCFP-EM program at U of T, which is a popular PGY3 program offered to family medicine residents. It has been demonstrated that participation in PGY3 training program is associated with increased participation of out-of-office care [46,47]. Interestingly, the more recent cohort of family medicine graduates were also more likely to do hospital visits than their counterparts in 2005/06, even though the inpatient experience of U of T FM trainees is limited in comparison to other medical schools in Ontario [48]. From 1993/96 to 2000/03 all FM led inpatient wards were closed in teaching hospitals in Toronto. This is also in contrast to a US study of a Missouri FM residency program which found a decline in the proportion of FP graduates performing hospital care [13]. In an Australia study, recent general practitioner (GP) graduates provided fewer services on average than previous GP graduates [30]. Fewer than 10% of FM graduates did obstetrics, nursing home visits or home visits. However, this is comparable to how all family physicians in Ontario are currently practicing [44]. Surveys from several FM residency programs have seen a decline in the more recent graduates providing obstetrical care [15,17].

Similar to other reported studies, female FM graduates appeared to carry less workload than their male colleagues [49,50]. However, there is some indication that the most recent female FM graduates did have a higher workload. With respect to physician resource planning, this trend should be watched given the proportion of women entering medical practice is increasing and now surpassing men [51]. An Australian study also demonstrated similar trends with an increasing female GP workforce and decrease in the male GPs working more than 11 sessions per work [11]. The workload measures used in this analysis are based on services billed. This is a proxy measure of workload and does not reflect the care provided to complex and higher acuity patients who take more time to manage. Taking care of sicker patients may lead to fewer services billed. Further analyses clearly measuring workload and its impact on primary care practitioners is indicated.

FPs are increasingly taking of care patients who are more ill [52]. The proxy measures in this study suggest that FM graduates take care of a high proportion of patients with high comorbidity. Training programs need to recognized the need be able to take care of complex patients. Policies developed for the retention of FPs need to also consider the workload and practice content of family medicine [53]. In addition, funding mechanism need also include measures of comorbidity to ensure more complex patients have equal access to primary care.

FM graduates are seeing a lower proportion of older patients that FPs practicing in the province. This may be a reflection of patients staying with their FPs and growing older as a practice matures. However, as the proportion of people over the age of 65 years increased over the next decade, along with the retirement of FPs in Ontario, FM graduates will need to take over the care of more seniors.

In 1997, an agreement between the Ontario Medical Association and the Ontario Ministry of Health discounted fees paid to new FPs practicing in areas deemed oversupplied [54]. The goal of this initiative was to encourage new FPs to practice in underserviced areas in the province. The impact of this policy initiative is evident in the fact that for the 1997 to 1999 cohort of FM graduates, there was a drop in the proportion practicing in the Toronto Central LHIN, which includes the City of Toronto and was at that time deemed an oversupplied area. In contrast, from 1997-1999 there was an increase in the proportion of FM graduates in the Central, Central West, South West and Erie St Clair LHINs. These trends were reversed in the subsequent time period after the policy to discount fees was removed.

This study used routinely collected health administrative data to measure the practice patterns and workload of FM graduates. These measures were able to described changes in FM graduates care over time and geography and this will contribute information towards health care planning. For example, while U of T FM graduates practice in all areas of the province, they are still more likely to practice close to the Toronto area. This is important information to support the development of FM training programs in more remote or underserviced areas of the province. In fact, this has occurred with the opening of the Northern Ontario School of Medicine in 2005 and the expansion FM training programs, such as the inclusion of the Barrie and Newmarket sites (deemed underserviced in Ontario) supported by U of T FM training program. These administrative data can be used to assess the impact of these programs in expanding FM care in remote or underserviced communities. This data also did demonstrate the effects of a policy of remuneration restrictions to influence the distribution of new FM graduates in Ontario.

We need to set up a system level ability to monitor physician supply, practice patterns and patient demand to be able to adjust enrolment increases and decreases into FM training. This involves more than just estimating a head count or number of FM graduates exiting programs, but also examining measures of their workload and productivity. Health administrative data in Ontario is routinely collected and accessible to researchers. In this study, we were able to use health administrative data measures of FM workload to describe time trends and changes by FP gender which can contribute to the planning of input into FM training.

These health administrative measures of practice patterns can also contribute information about the types of services provided by FPs for the planning regional health care needs. For example, local health regions need to know how FPs contribute to the supply of obstetrical services, emergency room care and the care of seniors and how attracting new FM graduates can contribute to this care. For post graduate medical programs, it is important to ensure they provide FM residents with the training appropriate to the changing patient population and acuity. This could include additional opportunities for enhanced training in geriatrics, musculoskeletal health and obstetrics.

In 2006, about 22% of FPs in Canada (and 24% in Ontario) used an Electronic Medical Record (EMR) as part of their clinical practice [55,56]. The information contained within an EMR could better describe the breadth of care provided by FPs. For example, non-billable work is documented within EMRs such as clinical communications to other health care providers, telephone care and supporting documentation for health care services. As the uptake of EMRs within FM improves and methods to extract EMR data for research purposes are developed in Canada [57], the information from EMRs can be used to describe care provided by FPs and other health care providers and further contribute to workload measures.

### Limitations

There are several limitations to our study. Patient socioeconomic status and comorbidity were based on proxy measures using administrative data. There may be some misclassification of patients using these proxy measures. Workload measures were based on physician consultation visits and they did not include telephone consultations and administrative work. This may not reflect the actual amount of work to take care of ill patients. We are unable to capture encounter data from CHC physicians. The work done by FM graduates working in a CHC will be underrepresented. We may also be underestimating work done by FPs who shadow bill within certain primary care models. Finally, only FM graduates from one medical school in Canada were examined.

## Conclusions

Routinely collected health administrative date can be used to measure FM productivity and workload. This information can contribute to improve planning of regional health care services, adjust intake numbers into FM training programs and feedback into the content of FM training programs. FM graduates from the largest Canadian medical school provide care to all areas of the province with increasing workloads and patient high comorbidity. More work is still need to evaluate more recently introduced initiatives to increase physician supply. Since 2000, there has been an expansion of the IMGs accepted into postgraduate training and therefore a need to address the impact of this program. New primary care models, decentralization of training and a new Northern Ontario School of Medicine will most likely also impact the care and distribution of new graduates. Finally this analysis should be extended across all 17 Canadian medical schools and take into account the various other routes of entry such as IMGs, re-entry and alternative routes to licensure.

## Competing interests

The authors declare that they have no competing interests.

## Authors' contributions

All the authors were involved in the conception and design of the study. SES was primarily responsible for the data analysis. RLJ and SES prepared the first draft of the article. CA, RHG and SV contributed to subsequent revisions. All of the authors reviewed the article and approved the final version.

## Pre-publication history

The pre-publication history for this paper can be accessed here:

http://www.biomedcentral.com/1471-2296/13/26/prepub
